# Fast Ecotoxicity Detection Using Biosensors

**DOI:** 10.1007/s11270-017-3341-5

**Published:** 2017-04-05

**Authors:** Martina Buckova, Roman Licbinsky, Vilma Jandova, Jan Krejci, Jana Pospichalova, Jiri Huzlik

**Affiliations:** 1 Transport Research Centre, Líšeňská 33a, 636 00 Brno, Czech Republic; 2 BVT Technologies, a.s, Strážek 206, 592 53 Strážek, Czech Republic

**Keywords:** Toxicity, Biosensor, Green algae, Oxygen electrode, Water and soil pollution, Silver nitrate

## Abstract

The article provides information about a new device, AlgaTox developed in the R&D project sponsored by the Technology Agency (n.TA02030179) and patented in Czech Republic (CZ 305687). Its functionality is based on the use of biosensor, and its main advantage is fast response rate. The toxicity detection is achieved through precise measurement of green algae oxygen production dynamics after their exposure to light of wavelength of 680 nm. Clark sensor with a resolution of 0.05% of the equilibrium oxygen concentrations and stability at a constant pressure and temperature of 0.1% of the equilibrium oxygen concentration at the 24-h measurement is used for the oxygen detection. Laboratory testing of the device has been made using silver nitrate, substance with known inhibitory effect on algae. Real samples of aqueous soil extracts and waste sample from old dried-up industrial tailing pond enriched with insecticide have been also tested. The values of oxygen production inhibition or stimulation determined with the new device in the evaluation of real samples were up to six times higher in comparison with the corresponding values of inhibition (stimulation) of growth rates determined by standard procedure.

## Introduction

The environmental pollution is currently at such level that the systematic control is almost impossible. Nevertheless, it is necessary to secure the quality of the environment for future generations. The monitoring of the environment is done using the classic chemical analyses and eco-toxicological testing. The advantage of biological tests is that they can determine the toxic effects of the sample on the sensitive organism. Its reaction allows a complete detection of the effects of chemical substances on the environment, and it is even possible to determine the toxic effects of those harmful substances, whose classic analysis has not been methodically done yet. The fact that the biological testing can detect mutual interaction of toxic substances in their mixture is an important advantage (synergetic or stimulatory effect, eventually mutual inhibition). These phenomena can be observed by classic analyses only with great difficulties. Plants and animals of various trophic levels are used for testing, and they have been chosen due to their sensitivity to chemical compounds and environmental pollution. The main disadvantage of these tests is that they are very time-consuming. They require several days for cultivation of organisms with toxic substance or environment sample. The information about the toxicity of the given sample is obtained after a longer period, and it does not provide a possibility for timely intervention in the case of positive toxicity test. These measurements can be simplified and accelerated with the use of biosensors. Biosensors are analytical instruments which use biochemically sensitive material for obtaining chemical information without the need for a complete processing of the sample. A suitable converter converts the output signal from the biosensor to quantitatively measurable signal. The most frequent output signals are current and voltage (Brayner et al. 2011).

A wide range of biosensors has been developed in the last decade; they have served for detection of herbicides, heavy metals, volatile organic substances, and even chemical weapons. They allow a quick measurement without complicated sample preparation. All these devices use biorecognition element (e.g., cells of algae, cyanobacteria, and microorganisms) immobilized in matrix, which prevents from its leaching without reduction in cell stability and activity (Brayner et al. 2011). In some cases, an active biological element, such as green algae photosystem II, is isolated and immobilized as the active element of the biosensor (Koblizek et al. 2002; Masojidek et al. 2011).

Microalgae which live in seawater and fresh water are very sensitive to the changes of their environment, and they allow detection of pollutants. For this reason, these photosynthetic microorganisms are used for a development of new biosensors (Brayner et al. 2011). The immobilization is the limiting step, because the materials used for immobilization have to be non-toxic for living cells and sufficiently stable (Lagarde and Jaffrezic-Renault 2011). Majority of the immobilization techniques is irreversible. The development in immobilization is heading towards the use of nanoparticles which would allow improvement of the biosensor sensitivity and stability. There are biosensors created by a combination of microbial cells and various nanomaterials (Sevcovicova and Tkac 2015). Nanomaterials can be used in construction of biosensors for monitoring of the environment (Durrieu et al. 2013). The immobilization of the algae *Chlorella pyrenoidosa* using magnetic nanoparticles has been described; it allowed for an effective and reversible immobilization without any influence on the cellular metabolism (Zamaleeva et al. 2011; Fakhrullin et al. 2010).

Biosensors using microalgae have usually been based on the measurement of fluorescence of the chlorophyll contained in chloroplasts (optic) or on tracking the development of photosynthetic oxygen (amperometric) (Brayner et al. 2011). The biosensors that are being developed have often been focused on the detection of specific group of harmful substances. A portable microalgae biosensor based on pulse amplitude modulation (PAM) has been developed; it is used for tracking the change of concentration of copper in drinking water tanks and pipelines (Peña-Vazquez et al. 2010). Also, biosensors used for detection of inorganic substance, e.g., copper (II), have been described (Alpat et al. 2007; Alpat et al. 2008). Fluorescent biosensor created by immobilization of cyanobacterium *Anabaena torulosa* on a cellulose membrane, and application of poly(2-hydroxethylmethacrylate) has been used for detection of heavy metals (Cu, Pb, Cd) and pesticides (Shing et al. 2013). The detection of mercury in the presence of silver ions has been allowed by biosensor which uses the immobilization of green algae *Chlorella* cells on the active surface of the glassy carbon electrode (Singh and Mittal 2012a). Amperometric biosensor which serves for the detection of biologically available ions of heavy metals, such as zinc, copper, cadmium, and nickel, has been produced using the algae *Chlorella* sp. fastened in polymerous membrane directly attached to the platinum electrode surface (Singh and Mittal 2012b). Microfluidic chip, based on the measurement of seawater algae mobility, has been used to determine the toxicity of heavy metals (Zheng et al. 2012). The green algae *Chlorella vulgaris* has been often used in the construction of biosensors designed for the detection of herbicides (Nguyen-Ngoc and Tran-Minh 2007; Shitanda et al. 2009; Rashkov et al. 2012) as well as for detection of organic substances, such as toluene and benzene (Shitanda et al. 2005). Biosensors using immobilized other algae species, both sea (Durrieu et al. 2011) and freshwater algae (Peña-Vazquez et al. 2009; Ferro et al. 2012; Husu et al. 2013), have been developed for herbicide monitoring.

Newly developed device AlgaTox is not focused on the detection of specific group of harmful substances; it determines the overall toxicity of the sample. As a biorecognition element, it uses green freshwater algae which are very sensitive and are also used in standard test described by the EN ISO 8692 (2012) standard. The algae are not immobilized, and they are used in a suspension. The measurement is not affected by the form of immobilization, and it is more reproducible and more robust. The device is based on extremely sensitive oxygen detection, which allows detection of oxygen production interference during photosynthesis due to the presence of pollutants within a few hours. The standard algae test (EN ISO 8692 2012) is based on a different principle—the measurement of the change in growth rate. Its reliable measurement takes at least 72 h. The acceleration of the analysis is the biggest benefit of the AlgaTox device. It allows a faster indication of the environmental pollution by preselecting potentially harmful samples which need an increased level of attention in a form of the standard ecotoxicology tests and chemical analyses. The device was patented in Czech Republic (CZ 305687).

## Materials and Methods

### Measurement Principle and Device Description

**Fig. 1:**
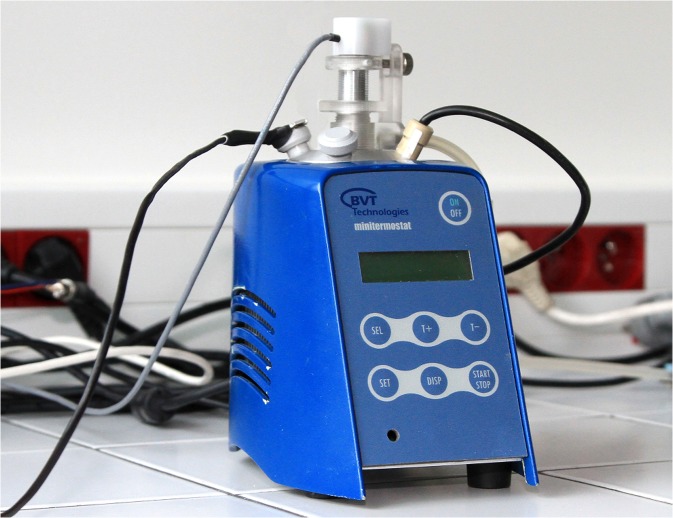
The AlgaTox device

The AlgaTox device (Fig. 1) measures the changes in green freshwater algae oxygen production for an indication of the environmental pollution. The algae are placed in the reaction vessel (3–5 mL) and illuminated with red light of a wavelength of 680 nm. This light is optimum for algae growth, respectively it corresponds to maximal light absorption by chlorophyll. LED is used as the source of light. The light source is fitted with a cone for its firm and tight insertion into the opening of the reaction vessel. Radiation is brought to the surface of the sample by a light guide. Light and darkness periodically alternate. One such alternation forms one light cycle. The exposure time and the time of the dark phase can be set in the program. The number of sample measurement cycles can be set as necessary. The reaction vessel does not allow access of ambient light. During the measurement, the algae are constantly stirred by stirrer (adjustable speed 10–1300 RPM).

The oxygen production is a signal of the biorecognition element of the biosensor, and it is detected by the oxygen electrode. The decline in the oxygen production is an indicator (chemical information) of toxic substances presence. The oxygen electrode is the Clark type electrode. It is composed of the electrode body, in which the glass body of the cathode is mounted; the cathode is made of platinum (Pt). The reference electrode made of silver (Ag) is placed on the glass body. Teflon membrane, which allows transfer of oxygen and some other gases (nitrogen), is placed on the active surface of the working electrode. The electrode cover with the Teflon membrane is screwed on the electrode body and filled with electrolyte (1 M KCl). The optimum distance between the stirrer and the electrode is 0.5–1 mm. The oxygen electrode is optimized in such a way, that its long-term stability (24 h) at a constant atmospheric pressure and constant temperature is better than 0.1% of equilibrium oxygen concentration in water at given conditions. The resolution of this oxygen measurement is better than 0.05% of the equilibrium oxygen concentration in water at given conditions. These parameters allow the device to accurately determine even small changes in the life cycle of algae. Thus, the high sensitivity of the toxicity determination is achieved.

**Fig. 2:**
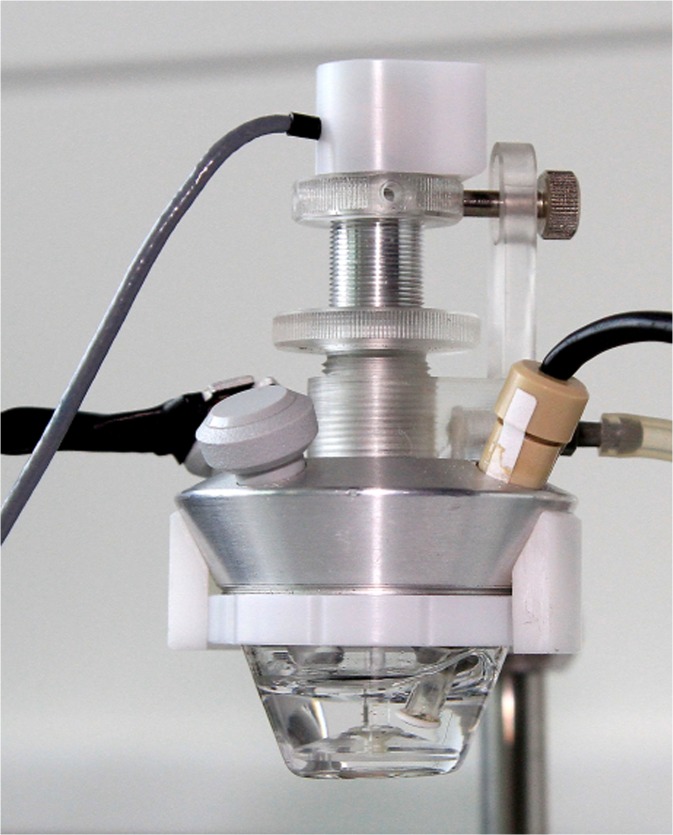
Reaction vessel detail

The reaction vessel detail is shown in Fig. 2. The central hole of the vessel is designed for the stirrer. The four smaller holes are designed for sample injection, oxygen electrode placement, the light source, and other accessories (aeration, other biosensors, photometer, stoppers). The reaction vessel is hermetically sealed with stoppers, and the standard atmosphere is ensured by washing with filtered air. During the measurement, the reaction vessel is placed in the copper thermoblock of the device. The temperature of the thermoblock is controlled by thermostat with Peltier element and kept in a range of −10 to +60 °C. The temperature in the reaction vessel stabilizes at the time < 10 min. Bioanalyzer (potentiostat) which serves as a convertor of the signal from the oxygen electrode is part of the device. The bioanalyzer allows basic operation with the measured data, e.g., data display, print or export to Excel/text file.

### Measurement Process and Data Evaluation

The amount of 5 mL of suspension of pre-cultivated algae has been pipetted into the reaction vessel. The measurements have been carried out with the algae *Scenedesmus quadricauda*, strain GREIFSWALD/15, from the Culture Collection of Autotrophic Organisms of the Institute of Botany of the Czech Academy of Science (CCALA, Třeboň, CZ). The algae intended for the measurements have been pre-cultivated in ½ Simmer-Šetlík medium (Zachleder and Setlik 1982) at the temperature of 24 ± 1 °C and illumination of 6000–7000 lx. At the beginning of the measurement, the algae cell density has been calculated under the microscope using the Bürker chamber. The optimum algae cell density for the measurement has been 700,000 to 1,500,000 cells per milliliter. The algae suspension has been tempered to the desired temperature at a constant stirring (set to 450 RPM) ca. 15 min before the measurement. After the oxygen concentration has settled, the algae illumination program has started. The experiments have been set to light cycles of alternating intervals of 300 s of light and 300 s of darkness. The oxygen production after illumination and the return to the steady state in darkness have been recorded. The measurement has consisted of five series of light cycle for measurement of the pure algae suspension. After five of these cycles, a sample or chemical compound has been pipetted for a 12-hour measurement of the oxygen production. Control measurement (i.e., algae suspension with no toxic substance added) was carried out for each set of data (i.e., for silver nitrate, aqueous extract of the soil, and aqueous extract of waste) under the same conditions. The length of the control measurement has been the same as for the test samples. The measured signal has indicated the actual oxygen concentration in the reaction solution. The average oxygen production and its time run have been calculated from the development of oxygen concentration during illumination.

In the case of real samples, where the toxicity was supposed to have been lower, it has been necessary to centrifuge the algae first in order to concentrate it. Five milliliters of medium has been pipetted into the reaction vessel, and concentrated algae has been added in such amount that the cell density in the vessel has been in range between 700,000 and 1,500,000 cells per millimeter. The suspension has been left stirring and tempering for the set temperature for 15 min, and then the measurement was started in the same way as described above. After 5 cycles, the vessel content has poured out, and it has been rinsed with the distilled water. The concentrated algae together with 5 mL of the sample were pipetted into the empty vessel, and after temperation to the set temperature, the oxygen production of the sample was measured.

The measured data has been exported to Excel, and a template for experiment evaluation has been compiled in the same program. The individual steps of the evaluation have been processed in separate sheets of the template whereas the order of sheets corresponded to the evaluation procedure. During the measurement, the data has been inserted in the first sheet. The evaluation has been then processed fully automatically. In the template, it has been possible to observe the overall course of the evaluation and the oxygen production time courses or to change the area in which the measured data was approximated, filtered, or displayed.

The *Y* value, which represents the relative decrease of algae oxygen production in course of the measurement related to its production at the beginning of the measurement, has been determined from the values of algae oxygen production. For every 12-hour measurement, last 10 of the *Y* values have been averaged. The average of the *Y* values for the tested samples and their concentration series have been related to the average *Y* value of the corresponding control measurement. The oxygen production inhibitions and stimulations for the tested samples have been calculated in this way. The EC_50_ values for all measurements have been calculated using the GraphPad Prism 6 software (GraphPad Software, Inc., USA).

### Tested Samples

The silver nitrate has been chosen for the laboratory verification of the device, as it is highly toxic for the green algae; the concentrations of 1, 0.8, 0.7, 0.6, and 0.4 mg L^−1^ have been used. The ½ Simmer-Šetlík medium (Zachleder and Setlik 1982) has been used for the preparation of concentration series. The device has been further tested with the real samples of aqueous extract of soils collected from the roadsides. The aqueous soils extract has been prepared in accordance with the Guidelines for the determination of toxicity of waste prepared by the Ministry of the Environment of Czech Republic (2007) and the EN 12457-4 (2002) standard. Due to the low toxicity of these samples, a waste sample from the inter-laboratory comparison 2015 (PT/TX/1/2015) has been chosen for further testing. Enriched sediment from an old, dried-up tailing pond of a metal processing industrial enterprise has been the sample. A waterproofing product—Insecticide Dřevosan Profi 058/14/506—has been used as a contaminant (CS lab spol. s r. o. 2015). An aqueous extract from this sample has been prepared in the same manner as for the soil samples. Dilution series of an aqueous extract of the waste in concentrations 1000, 800, 600, 400, 200, 100, and 10 mL L^−1^ have been prepared for the measurements in AlgaTox.

The classic algae tests according to EN ISO 8692 (2012) standard have been carried out for all samples tested in the AlgaTox device. Stimulation of growth during performance of the standard algal test has been observed in most of the real samples of aqueous soil extracts. Soil samples that exhibited both inhibition and stimulation of growth rate in the standard algae test were selected for the measurements in AlgaTox.

## Results and Discussion

The algae oxygen production has been measured and recorded in the AlgaTox device for all test samples and the control sample. The algae are very sensitive to the environmental pollution, they are of suitable size, and their life cycle is well-known. They are used in the test described in the EN ISO 8692 (2012) standard as well. The standard allows the use of various kinds of algae. Algae *Pseudokirchneriella subcapitata* and *Scenedesmus subspicatus* (both recommended by the EN ISO 8692 (2012) standard), *Chlorella vulgaris* (often used in biosensors, Nguyen-Ngoc and Tran-Minh 2007; Shitanda et al. 2009; Rashkov et al. 2012; Singh and Mittal 2012a, b) and *S. quadricauda* (used in an earlier version of the standard) have been tested in the AlgaTox device. The algae *S. quadricauda* has been chosen based on the process of measurement conditions optimization. These algae have showed the highest stability of the measured signal and at the same time have given a signal strong enough for a further processing in comparison with other tested species of algae that showed significantly smaller signal and high noise values on the contrary.

Unlike in the standard algae test (EN ISO 8692 2012), these measurements have been carried out at 33 °C. This temperature has been set by experimental optimization of the system sensitivity. At this temperature, the reaction of algae is the fastest (faster diffusion), and the oxygen electrode signal is the highest. The oxygen electrode signal rises at higher temperatures, but the algae oxygen production decreases due to excessively high temperature.

### Silver Nitrate

**Fig. 3:**
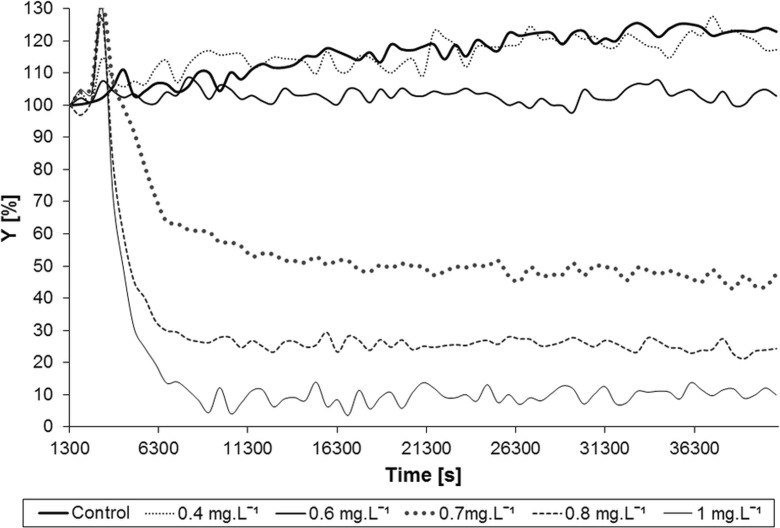
Results of 12-hour measurement of silver nitrate using the AlgaTox device (the *Y* value represents the relative decrease of algae oxygen production in course of the measurement related to its production at the beginning of the measurement)

**Fig. 4:**
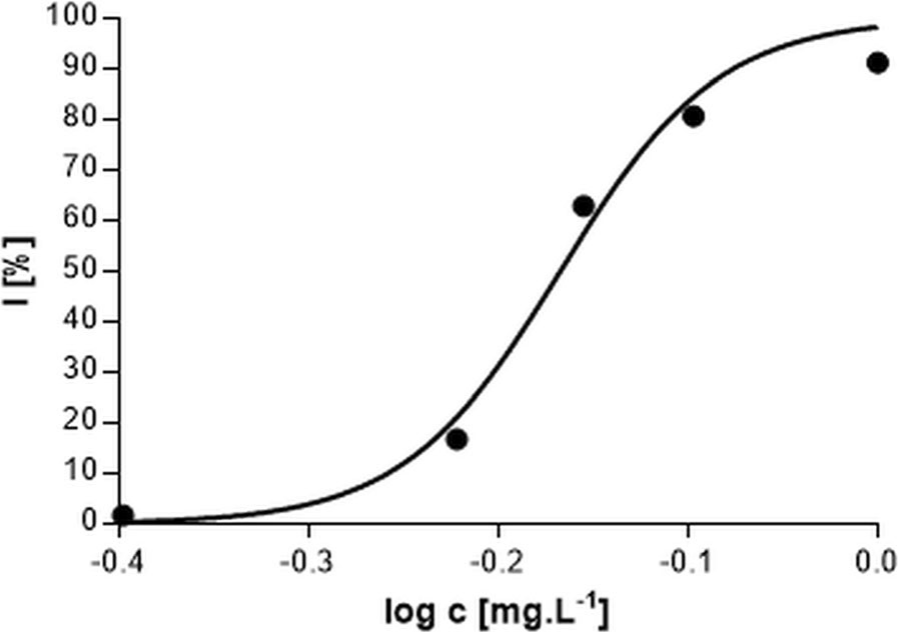
Response curve of the dependency of oxygen production inhibition on the silver nitrate concentration

The results of 12-hour measurement for the individual concentrations of silver nitrate using the AlgaTox device are shown in Fig. 3. The response curve of the dependency of the oxygen production inhibition on silver nitrate concentration is shown in Fig. 4. From the results in Fig. 3, it is clear that for the concentration of 0.4 mg L^−1^ in course of the 12-h measurement, a slight decrease in the oxygen production occurred in comparison with the control population (2%). For the concentration of 0.6 mg L^−1^, the oxygen production decreased for approximately 20%. For concentrations higher than 0.7 mg L^−1^, a significant decrease in the oxygen production occurred within the first hour of the measurement. The EC_50_ value for silver nitrate has been determined by the standard algae test according to the EN ISO 8692 (2012) standard to be 0.076 mg L^−1^. The EC_50_ value for the AlgaTox device has been 0.68 mg L^−1^, which is nearly 10 times higher. After 12 h of measurement, the device has been able to detect concentration approximately six times higher than the EC_50_ value for the standard algae test using silver nitrate.

### Real Samples of Aqueous Soils Extracts

**Fig. 5:**
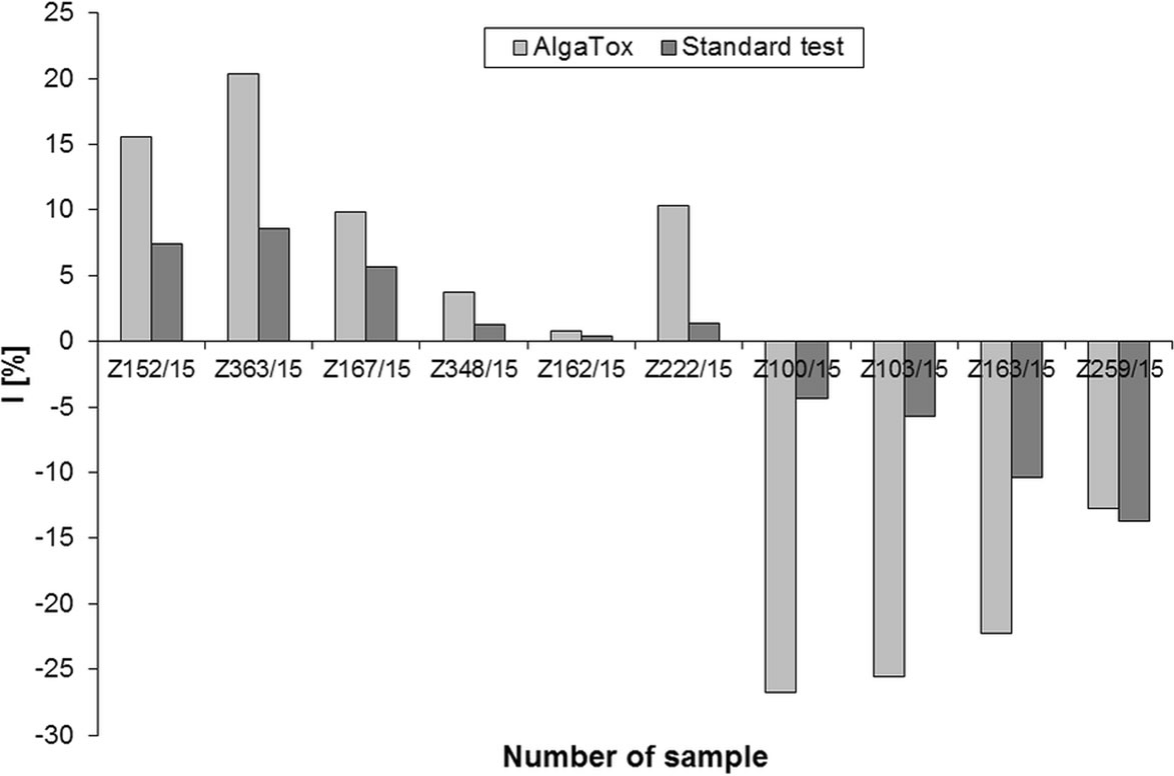
Results of measurements of the real samples using the AlgaTox device and comparison with standard algae test

In the graph in Fig. 5, there are results of the testing of selected real samples of aqueous extract of soils. The values of inhibitions (stimulations) of the algae oxygen production as determined by the AlgaTox device are compared with values of inhibitions (stimulations) of growth rates determined by the standard procedure (EN ISO 8692 2012). The values of the oxygen production inhibition for four samples have been up to two times higher than the values of growth rates inhibitions determined for the same samples by the standard procedure (EN ISO 8692 2012). The results of the two methods have been comparable in two cases, where the values of inhibitions approached zero. The samples showing the stimulation of the growth rate have showed stimulation of the oxygen production as well. The differences between the two methods have been more distinctive in the case of stimulations than in the case of inhibitions. As shown in Fig. 5, when comparing the toxicity of selected samples using the two methods, the trend is the same. In comparison with the standard algae test, higher values of oxygen production/inhibitions and stimulations have been found, except for the sample Z259/15 where the value of oxygen production/stimulation has been lower than the value of the growth rate stimulation.

### The Waste Sample from Inter-Laboratory Comparison

**Fig. 6:**
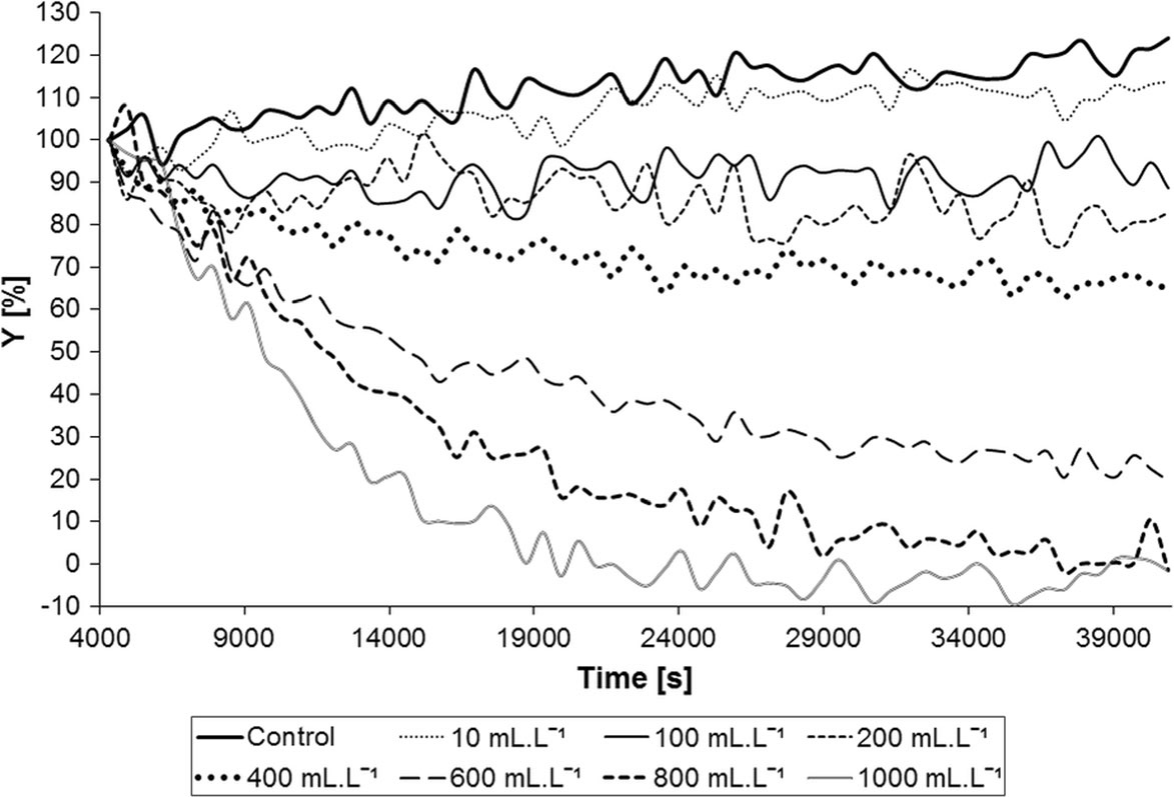
Results of 12-hour measurement on aqueous waste extract using the AlgaTox device (the *Y* value represents the relative decrease of algae oxygen production in course of the measurement related to its production at the beginning of the measurement)

**Fig. 7:**
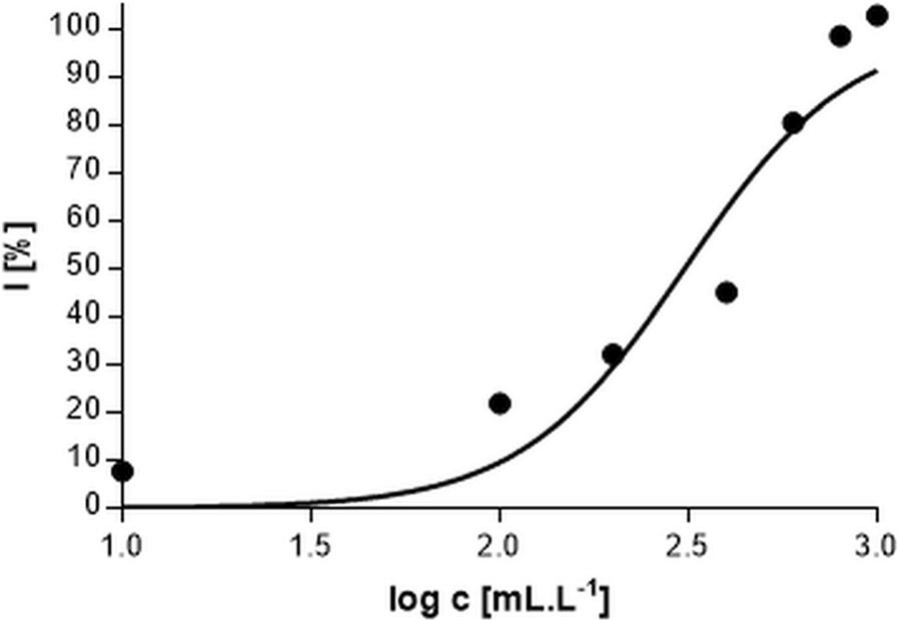
Response curve of the dependency of oxygen production inhibition on the concentration of aqueous waste extract

The results of 12-hour measurement for the individual concentrations of the aqueous waste extract using the AlgaTox device are shown in Fig. 6. The oxygen inhibitions have been calculated for each concentration. The values of inhibitions obtained in this way serve for calculation of the EC_50_ value and construction of the response curve of the dependency of oxygen production inhibition on the concentration of aqueous waste extract (Fig. 7). The EC_50_ value for AlgaTox and for the aqueous waste extract has been 309.8 mL L^−1^. The EC_50_ value for the waste extract determined by the algae test according to the EN ISO 8692 (2012) standard has been 8.91 mL L^−1^. The reference value determined from the results of the inter-laboratory comparison has been 10.68 mL L^−1^ (CS lab spol. s r. o. 2015). The EC_50_ value for the AlgaTox device has been approximately 30 times higher than the EC_50_ value set for the standard algae test. In comparison with the control population, a decrease of 10% in the oxygen production has been observed at the concentration of 10 mL L^−1^ and of 30% at the concentration of 100 mL L^−1^. The advantage of the AlgaTox device is that it tracks the dynamics of the process. That has allowed detection of high concentrations within 20 minutes (for 1000 mL L^−1^ the determinable decrease is 10% of the signal).

The decrease area of 0–100% corresponds to the direct inhibition of the photosystem II. For these concentrations, a full inhibition of the growth rate in the standard algae test can be expected. Relevant comparison of tests according to EN ISO 8692 (2012) standard and test using the AlgaTox device can be done only in the area of 100–125%, where the device detects growth rate on the basis of the oxygen production (the oxygen production increases with the growth of the size of the oxygen-producing colony). Currently detected growth rate of the control algae population is *λ* = 1.59 × 10^−2^ h^−1^ (4.42 × 10^−6^ s^−1^).

The advantage of the AlgaTox device is that it works fully automatically. The 12-hour measurement in the automatic mode is very effective. It can be started in the evening, and the results are known in the morning. Despite the fact that the AlgaTox device does not reach the EC_50_ values specified in the standard procedure (EN ISO 8692 2012), the acceleration of the measurement is crucial. In cases of great need for fast determination of the sample toxicity, the difference between 12 h and 1 to 2 weeks in gaining the results is very significant.

The experiments have proven that accurate measurement of algae oxygen production can be a fast preliminary alternative method of ecotoxicity determination; the detection limit is currently up to 10 times higher than the EC_50_ of standard algae test; however, the results can be achieved within 12 h.

## Conclusions

The AlgaTox device brings a new method which is similar to classic toxicity test defined by the EN ISO 8692 (2012) standard. It allows measurement of integral influence of the pollution to the environment, resp. its impacts on living organisms. It is faster (ca. several hours vs. 72 h) and more accurate due to its algorithmisation and integration into the developed device. The main advantage of AlgaTox is its ability to detect toxic substances in high concentrations within hours. Another advantage is the possibility to continuously track, record, and evaluate the changes in the oxygen production in course of the whole measurement.

The EC_50_ values for the aqueous waste extract and for silver nitrate determined by the two methods are not comparable, and in the case of the aqueous waste extract are up to 30 times higher when using AlgaTox. These differences can be caused by different principle of the measurements; in the standard algae test, the growth rate inhibition is measured, while the AlgaTox device measures oxygen production, which is solely influenced by the toxic substances influencing the photosynthesis.

It has been proven that when testing real samples of a low toxicity, both methods have determined whether inhibition or stimulation occur in the selected samples. Nevertheless, the values of inhibition or stimulation of the oxygen production were up to six times higher than corresponding values of the growth rate inhibition (stimulation).

The device and its methodology have a much wider scope of application than just the detection of toxic substances in the environmental samples. The device can be used in chemical productions, resp. chemical substances evaluation in correspondence with the REACH legislation, where the producer can test the impact of chemicals on the living organisms. The device can be used for more than just the evaluation of the impact on algae but for accurate measurement of biochemical oxygen consumption, for accurate measurement of chemical oxygen consumption, for cell growth tests of tissues, small animal who consume or produce oxygen (respirometry), for sterility tests based on tracking of the growth of microorganisms or for the measurement of slow reaction rates with changing concentration of oxygen (e.g., enzyme activity of oxidase).
